# Urinary iodine concentration and thyroid function in pregnant women with hypertensive disorders

**DOI:** 10.3389/fendo.2025.1578597

**Published:** 2025-09-08

**Authors:** Adriana Duarte Miranda Queiroz, Maria Roseneide dos Santos Torres, Luana Cristina Fernandes Ratis, Maria Clara Vieira Morais, José Natal Figueiroa, Alex Sandro Rolland Souza

**Affiliations:** ^1^ Department of Endocrinology and Metabology, Federal University of Campina Grande (UFCG), Campina Grande, Paraíba, Brazil; ^2^ Instituto de Medicina Integral Prof. Fernando Figueira (IMIP), Recife, Pernambuco, Brazil; ^3^ Department of Gynecology and Obstetrics, Universidade Católica de Pernambuco (UNICAP), Recife, Pernambuco, Brazil; ^4^ Department of Gynecology and Obstetrics, Federal University of Pernambuco (UFPE), Recife, Pernambuco, Brazil

**Keywords:** iodine deficiency, gestational hypertension, iodine, pre-eclampsia, thyroid

## Abstract

Pregnant women are considered a risk group for iodine deficiency, and this condition may be associated with changes in thyroid hormone levels and an increased risk of developing hypertensive disorders.

**Objective:**

To assess urinary iodine concentration (UIC), thyroid-stimulating hormone (TSH) and anti-thyroid peroxidase (TPO) antibody levels in pregnant women with hypertensive disorders.

**Methods:**

This cross-sectional study was conducted in a referral maternity hospital in Paraíba, Brazil between June 2022 and April 2023. A total of 250 women over 18 years of age, in the third trimester of pregnancy and diagnosed with hypertensive disorders were included. Clinical and epidemiological data, as well as TSH, anti-TPO antibodies and UIC were assessed. Iodine deficiency was defined as UIC levels <150 μg/L. Correlations between UIC and the study variables were analyzed using regression models adjusted with the *cenreg* function of the Nondetects and Data Analysis for Environmental Data (NADA) statistical package.

**Results:**

The median UIC was 82.5 μg/L (95%CI: 72.9–93.8), with 76.4% of participants (n=191) being iodine deficient. UIC differed among hypertensive disorders (p=0.023), with significant differences found between gestational hypertension (111.6 μg/L) and both preeclampsia (61.3 μg/L; p=0.018) and superimposed preeclampsia (70 μg/L; p=0.020). Systolic blood pressure (SBP) and gestational age correlated negatively with UIC (-0.006±0.002, p=0.015; -0.03±0.01, p=0.042, respectively). No correlation was found between UIC and TSH levels or anti-TPO antibodies.

**Conclusion:**

Lower UIC levels were observed in pregnant women with pre-eclampsia or superimposed pre-eclampsia compared to women with gestational hypertension, as well as in higher SBP levels and gestational age. Detection of iodine deficiency in pregnancy may help identify women at higher risk of hypertensive complications, and supplementation may potentially improve outcomes.

## 1 Introduction

Iodine deficiency in pregnancy, defined as a urinary iodine concentration (UIC) <150 μg/L, affects 16.1% to 84.0% of pregnant women (1, 2). The potential adverse effects of severe iodine deficiency during pregnancy are primarily mediated by maternal thyroid dysfunction, as iodine is essential for thyroid hormone synthesis (3). These hormones are crucial for fetal development, with the fetus being entirely dependent on maternal thyroid hormones until the 12^th^ week of pregnancy (4). Furthermore, clinical hypothyroidism has been linked to an increased risk of pregnancy complications, including miscarriage, preeclampsia, gestational hypertension, placental abruption, postpartum hemorrhage, prematurity, low birthweight and elevated perinatal morbidity and mortality (5–8).

Hypothyroidism secondary to persistent iodine deficiency is believed to reduce endothelial nitric oxide and prostacyclin production, which promotes the upregulation of endothelin, leading to endothelial dysfunction and systemic vasoconstriction (9). Additionally, as iodine has antioxidant properties, its deficiency may increase oxidative stress, contributing to complications such as hypertensive disorders of pregnancy (10). Reduced iodine levels may also predispose women to preeclampsia by decreasing the antioxidant capacity of the placenta, an organ with high iodine concentration, thereby increasing oxidative stress and free radical production, which in turn exacerbate placental endothelial dysfunction (9, 10). Iodine deficiency is highly prevalent during pregnancy (11–13), particularly in women with hypertensive disorders (10, 14, 15).

The latest population study conducted in Brazil, with the aim of assessing the impact of salt iodization in the country, showed that the northeast region had the highest median CUI (298.80 μg/L) (16). However, given the higher prevalence of iodine deficiency in pregnant women with hypertension and its potential link to an increased risk of preeclampsia, this study aimed to assess iodine status, the prevalence of iodine deficiency, and its association with maternal thyroid and autoimmune function in a population of pregnant women with hypertensive disorders at a referral maternity hospital in northeastern Brazil.

## 2 Materials and methods

This cross-sectional study assessed third-trimester pregnant women with hypertensive disorders between June 2022 and April 2023 at a maternity hospital in northeastern Brazil. Exclusion criteria consisted of disorders of intestinal absorption, previous thyroid disease, psychiatric conditions and use of levothyroxine, antithyroid agents, multivitamins or drugs containing iodine. The presence of previous illnesses was obtained through self-reports from the participants. Sample size was calculated using EpiInfo StatCalc (version 7.2), for a 95% confidence level, an expected margin of error of 5% and an estimated iodine deficiency prevalence of 80%, obtained through a pilot study conducted with the first 100 pregnant women in the sample. The calculation showed that a minimum of 250 participants were required for the study.

The study sample consisted of pregnant women who were hospitalized at the time of the research, and therefore, the collection of CUI was restricted to the third trimester of pregnancy only.

The numerical variables evaluated included maternal age (years), gestational age at UIC sampling (weeks), number of pregnancies, and body mass index (BMI) (kg/m^2^). Maternal age was categorized into three groups: < 25 years, between 25 and 34 years, and ≥ 35 years (17, 18). Gestational age at UIC sampling was categorized into preterm (< 37 weeks) or term (≥37 weeks). Number of pregnancies was divided into one previous pregnancy or ≥2 pregnancies. BMI was classified according to the Atalah curve: low weight, adequate weight, overweight or obese (19). Categorical variables included ethnicity (white or brown/black); having a partner (yes or no); place of residence (*Agreste* or *Borborema/Sertão*); schooling (illiterate/elementary or high school/university); income (yes or no); smoking (yes or no); alcohol consumption (yes or no); use of illicit drugs (yes or no); previous diabetes mellitus (DM) or gestational DM (yes or no); and type of hypertensive disorder (20).

Pregestational DM was defined as type 1 or type 2 DM prior to conception, or fasting glucose ≥126 mg/dl during pregnancy. Gestational DM was defined as abnormal fasting glucose or an abnormal oral glucose tolerance test (OGTT) first registered at any point during pregnancy. In the first trimester, normal fasting glucose levels <92 mg/dl led to recommendations to perform a 75-g OGTT at 24–28 weeks of pregnancy. Fasting glucose of 92–125 mg/dl was indicative of gestational DM. In the second trimester, fasting glucose ≥92 mg/dl and/or 1-hour glucose ≥180 mg/dl and/or 2-hour glucose ≥153 mg/dl during OGTT indicated gestational DM (20). Hypertensive disorders were defined according to the criteria of the International Society for the Study of Hypertension in Pregnancy as chronic hypertension, preeclampsia, eclampsia, HELLP (Hemolysis, Elevated Liver enzymes, Low Platelets) syndrome, superimposed preeclampsia with chronic hypertension, and gestational hypertension (20).

UIC (μg/L) was measured from a single urine sample (approximately 30 ml), collected in the morning or afternoon after a minimum two-hour interval since the last urination, with no visible vaginal bleeding. The samples collected in a universal collection container were transferred to a monovette tube (Nümbrecht, Germany) for transportation and storage at 2-8°C until analysis. Inductively coupled plasma mass spectrometry was performed with the Thermo ICAP-RQ analyzer (Bremen, Germany) in a single laboratory (Cerba-LCA, São Paulo, Brazil). The detection limit was 40.6 μg/L. UIC was categorized based on international criteria, with iodine deficiency defined as UIC <150 μg/L (1). TSH and anti-thyroid peroxidase (TPO) antibody levels were measured from 5-ml blood samples collected in the morning, transported under refrigeration (2-8°C) and analyzed by chemiluminescence. TSH reference values were 0.1-3.0 mIU/L, with values >4.0 mIU/L indicating hypothyroidism (7). Anti-TPO values ≥9.0 IU/ml were considered positive, in accordance with the laboratory method used.

Data analysis was performed using Epi-Info, version 7.2 (CDC, Atlanta, DC, USA) and R (Vienna, Austria, 2024). Frequency distribution tables were created for categorical variables. Medians were used to describe UIC, as the variable was asymmetrically distributed and left-censored for values <40.6 μg/L. Median UIC was compared using bootstrap resampling. The Benjamini & Hochberg method was used to adjust for multiple comparisons in the analysis of the types of hypertensive disorder.

Correlations between UIC and clinical/laboratory variables were calculated in regression models adjusted with the cenreg function of the Nondetects and Data Analysis for Environmental Data (NADA) package. Significance was set at p<0.05.

The study was approved by the internal review board of the *Instituto de Medicina Integral Prof. Fernando Figueira* (IMIP) under reference CAAE 58309422.7.0000.5201 and approval letter 5.443.633, dated June 1, 2022. Patients were included after providing informed consent.

## 3 Results

**Figure 1:**
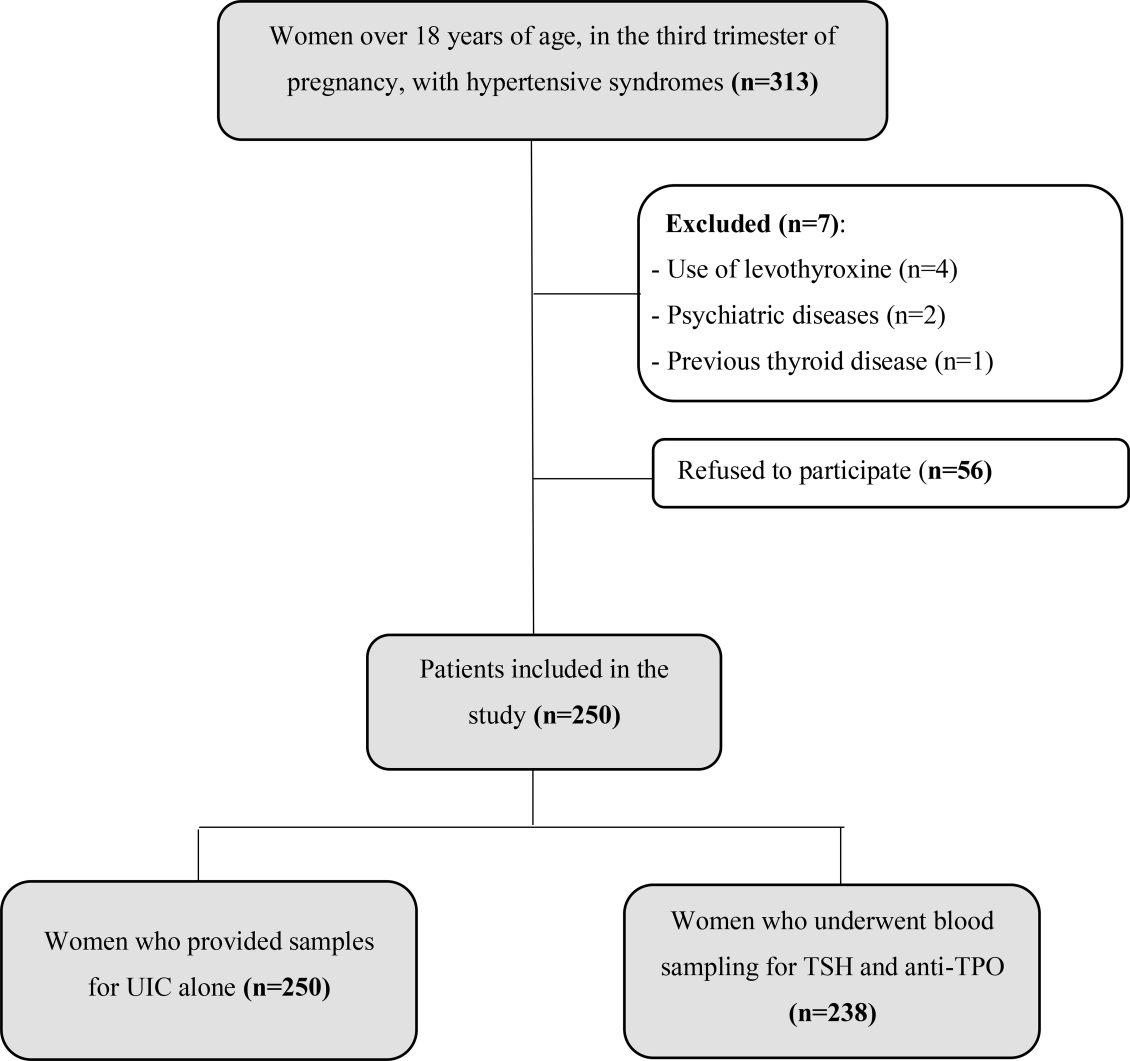
Flow chart of the enrollment of participants to the study. UIC, urinary iodine concentration; TSH, thyroid-stimulating hormone; anti-TPO, anti-thyroid peroxidase antibody.

A total of 313 pregnant women were screened. Seven were excluded due to previous thyroid disease, psychiatric conditions or use of levothyroxine. Another 56 women declined to participate, leaving 250 who provided urine samples for UIC analysis. Twelve of these women, however, refused to provide blood samples for TSH and anti-TPO analysis (**Figure 1**).

Of the 250 participants, 76.4% (n=191) had UIC <150 μg/L, with 14.0% (n=35) having UIC below the detection limit of 40.6 μg/L. Most women were 25–34 years of age (46%; n=115), had had ≥2 pregnancies (69.6%; n=174), self-identified as brown-skinned/black (58%; n=145), had a partner (81.6%; n=204), earned an income (52.2%; n=109), lived in an urban area (76.8%; n=192), resided in the Agreste region of Paraíba (85.2%; n=213), had high school or university education (51.3%; n=121), were obese (51.6%, n=129), did not have DM (60.4%; n=151) and did not smoke (99.2%; n=248) or drink alcohol (98.8%; n=247). No illicit drug use was reported (**Table 1**).

**Table 1:** Median urinary iodine concentration (UIC) (in μg/L) according to the clinical, epidemiological and laboratory characteristics of the women in the study.

Characteristics	n	%	UIC <150 μg/L (%)	Mediana (Q1-Q3)	95%CI	p-value*
Maternal age (years)						0.051
< 25	67	26.8	29.3	68.8 (45.2 – 115.4)	55.7– 79.6	
25 to 34	115	46.0	44.0	87.4 (56.2 – 151.0)	69.4– 104.2	
≥ 35	68	27.2	26.7	90.1 (54.8 – 149.3)	73.8 – 115.5	
Number of pregnancies						0.087
1≤1	76	30.4	31.9	71,9 (45,3 – 129,4)	61.3 – 88.0	
>1	174	69.6	68.1	86,0 (50,8 – 145,4)	76.4 – 105.8	
Gestational age at urine sample collection (weeks)						0.060
< 37	175	70.0	67.0	87,1 (53,0 – 161,3)	76.9 - 103.0	
≥ 37	75	30.0	33.0	65,6 (44,8 – 117,8)	59.1 - 85.8	
Area of residence						0.665
Urban	192	76.8	76.4	85,1 (50,9 – 149,2)	72.1 - 99.9	
Rural	58	23.2	23.6	78,4 (51,8 – 122,2)	63.3 - 102.5	
Ethnicity						0.701
White	105	42.0	41.4	85,6 (54,1 – 149,0)	72.7 - 108.8	
Black/Mixed-race	145	58.0	56.6	80,2 (50,2 – 138,6)	65.6 - 92.5	
Diabetes mellitus						0.480
Gestational or previous DM	99	39.6	36.1	85,8 (55,0 – 152,2)	73.1 - 121.6	
No	151	60.4	63.9	78,8 (46,0 – 129,5)	68.4 - 93.4	
Systolic blood pressure						0.490
≥160 mmHg	75	30.0	30.8	73,1 (45,0 – 139,1)	61.1-100.8	
<160 mmHg	175	70.0	69.2	85,8 (56,4 – 14,9)	75.3-100.4	
Diastolic blood pressure						0.168
≥110 mmHg	12	4.8	6.3	53,1 (40,6 – 97,7)	40.6-97.7	
<110 mmHg	238	95.2	93.7	85,0 (52,5 – 149,6)	74.5-95.0	
TSH ≥4.0 mIU/L						0.671
Yes	12	5.0	3.8	96,6 (46,1 – 205,8)	46.1-211.8	
No	226	95.0	96.2	80,7 (51,0 – 145,7)	71.1-94.1	
Anti-TPO antibodies						0.206
Positive	11	4.6	4.1	150,2 (61,7 – 174,5)	61.8 – 174.8	
Negative	227	95.4	95.9	79,6 (50,2 – 145,4)	69.5 – 93.4	
Smoking						0.762
Yes	2	0.8	1.1	72,6 (68,8 – 76,3)	–	
No	248	99.2	98.9	83,8 (50,9 – 147,9)	73.1-94.3	
Alcohol consumption						0.851
Yes	3	1.2	1.1	59,2 (58,0 – 152,3	–	
No	247	98.8	98.9	83,1 (50,8 – 146,8)	73.3-94.1	
Body mass index (kg/m^2^) **						0.132
Low weight/adequate weight^†^	64	25.6	27.7	70,1 (42,8 – 117,6)	55.7-89.9	
Overweight	57	22.8	23.6	77,0 (51,0 – 121,6)	64.5-92.4	
Obese	129	51.6	48.7	95,8 (58,4 – 153,2)	75.5-115.5	

95% CI, 95% confidence interval; DM, diabetes mellitus; anti-TPO, anti-thyroid peroxidase. ^†^Only two patients were classified as having low weight. *Bootstrap resampling method. **Difference in medians: Low/Adequate weight versus Overweight: -8.2 95%CI: -26.6 to 16.9, p=0.545; Low/Adequate weight versus Obesity: -9.3 95%CI: -29.6 to 9.6, p=0.298; Overweight versus Obesity: -1.1 95%CI: -24.2 to 14.3, p=0.687.

Urine samples for UIC were collected between 28 and 36 weeks of pregnancy in most cases (70%; n=175), with the median UIC being indicative of iodine deficiency in all the groups except for the group of women who tested positive for anti-TPO antibodies. The lowest median CUI was found in pregnant women under the age of 25 (68.8 μg/L; 95% CI: 55.7–79.6 μg/L). In most comparisons, no statistically significant differences were found in median UIC (**Table 1**).

The mean TSH level was 1.49±1.13 mIU/L and 95.4% (n=227) of the women had anti-TPO levels <9.0 IU/ml (negative). Of the women who tested positive for anti-TPO antibodies, only 1 (0.4%) had TSH > 4 mIU/L (5.74 mIU/L).

**Figure 2:**
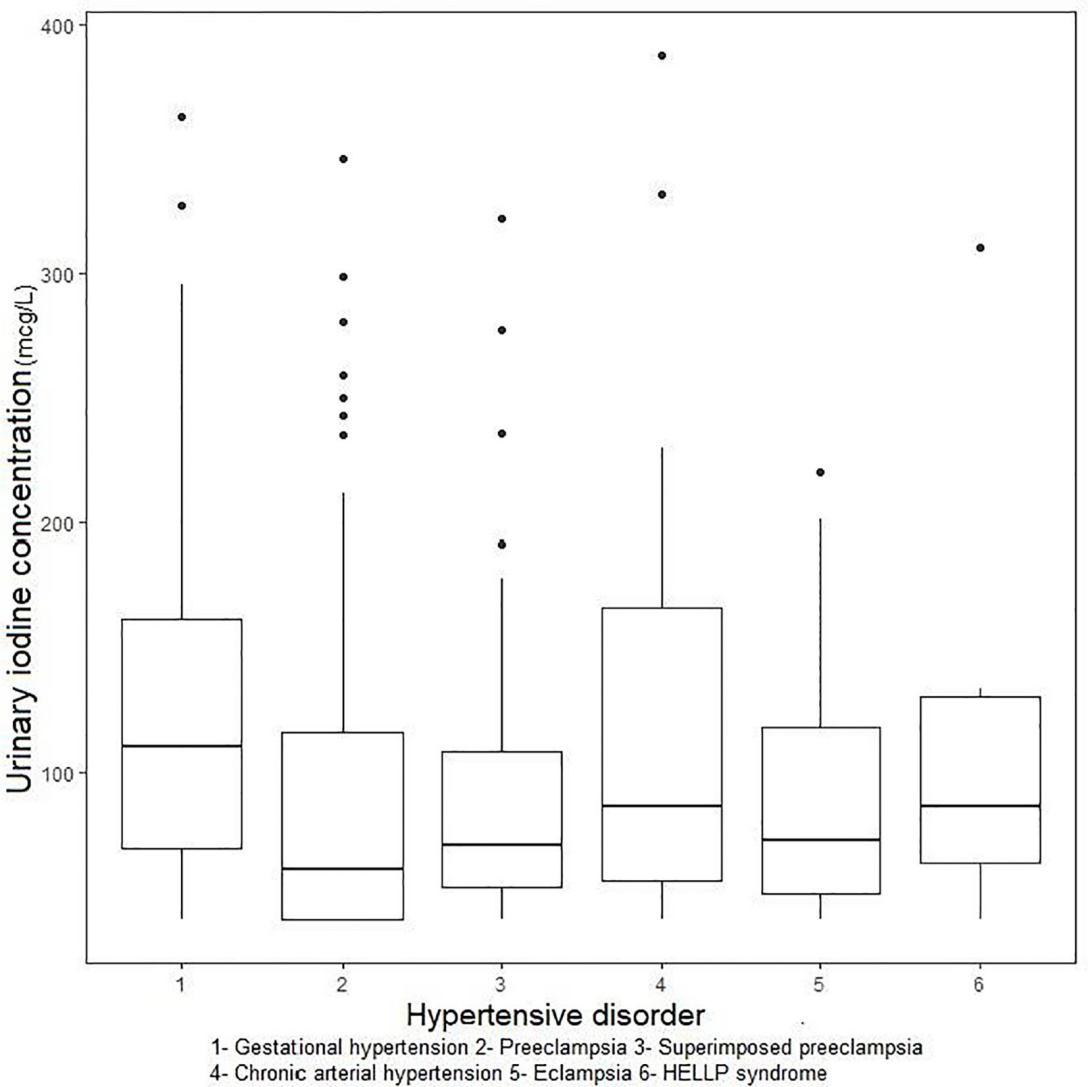
Urinary iodine concentration (UIC) medians according to type of hypertensive syndrome (μg/L).

The overall median UIC was 82.5 μg/L (95%CI: 72.9-93.8 μg/L). The lowest median UIC was found in women with preeclampsia (61.3 μg/L; 95%CI: 49.2-84.5 μg/L) and superimposed preeclampsia (70.1 μg/L; 95%CI: 64.0-83.1 μg/L). A statistically significant difference in median UIC was observed across types of hypertensive disorders (p=0.023) (**Table 2**, **Figure 2**).

**Table 2:** Median urinary iodine concentration (UIC) (in μg/L) according to type of hypertensive disorder.

Hypertensive disorder	n	%	UIC <150 μg/L (%)	Median UIC*	95%CI
Gestational hypertension	78	31.2	66.7	111.6	93.4 – 131.1
Preeclampsia	80	32.0	81.3	61.3	49.2 – 84.5
Superimposed preeclampsia	40	16.0	87.5	70.1	64.0 - 83.1
Chronic hypertension	29	11.6	69.0	86.3	68.1 - 145.8
Eclampsia	15	6.0	80.0	72.7	52.5 - 103.0
HELLP syndrome	8	3.2	87.5	80.2	46.0 - 133.9
**Total**	**250**	**100**	**100**	**81.2**	**73.1 – 94.1**

95% CI, 95% confidence interval. HELLP syndrome: Syndrome of hemolysis, elevated liver enzymes, and low platelet count. *Medians and their respective confidence intervals were obtained using the bootstrap resampling method (p=0.023).

Paired comparisons of UIC between hypertensive disorder categories showed statistically significant differences only between gestational hypertension and preeclampsia (Δmed 49.3 μg/L; 95%CI: 20.4-74.9 μg/L; p=0.018) and between gestational hypertension and superimposed preeclampsia (Δmed 39.9 μg/L; 95%CI: 16.8-64.4 μg/L; p=0.020) (**Table 3**).

**Table 3:** Comparison of urinary iodine concentration (UIC) medians between pairs of types of hypertensive disorder in pregnancy.

Comparison between groups of hypertensive disorders in pregnancy	Δ IUC Medians (95%CI)	p-value*	Adjusted p-value^**^
Gestational hypertension versus preeclampsia	49.3 (20.4 - 74.9)	0.001	0.018
Gestational hypertension versus superimposed preeclampsia	39.9 (16.8 – 64.4)	0.003	0.020
Gestational hypertension versus chronic hypertension	24.2 (-35.1 – 57.7)	0.555	0.640
Gestational hypertension versus eclampsia	37.8 (-15.9 – 70.8)	0.129	0.409
Gestational hypertension versus HELLP syndrome	24.2(-28.4 – 68.7)	0.448	0.640
Preeclampsia versus superimposed preeclampsia	-9.4 (-27.2 – 15.4)	0.312	0.639
Preeclampsia versus chronic hypertension	-25.1 (-84.1 – 8.4)	0.069	0.347
Preeclampsia versus eclampsia	-11.5 (-56.2 – 20.3)	0.478	0.640
Preeclampsia versus HELLP syndrome	-25.1 (-75.8 – 17.8)	0.201	0.503
Superimposed preeclampsia versus chronic hypertension	-15.7 (-76.2 – 10.7)	0.136	0.409
Superimposed preeclampsia versus eclampsia	-2.1 (-44.6 – 24.9)	0.861	0.889
Superimposed preeclampsia versus HELLP syndrome	-15.7 (-65.8 – 22.1)	0.366	0.639
Chronic hypertension versus eclampsia	13.6 (-51.5 – 71.5)	0.384	0.639
Chronic hypertension versus HELLP syndrome	0.0 (-72.2 – 64.6)	0.889	0.889
Eclampsia versus HELLP syndrome	-13.6 (-76.0 – 45.2)	0.552	0.640

HELLP syndrome: Syndrome of hemolysis, elevated liver enzymes, and low platelet count; Δ: difference.

^*^Bootstrap resampling method; ^**^Benjamini & Hochberg method for multiple comparisons.

**Figure 3:**
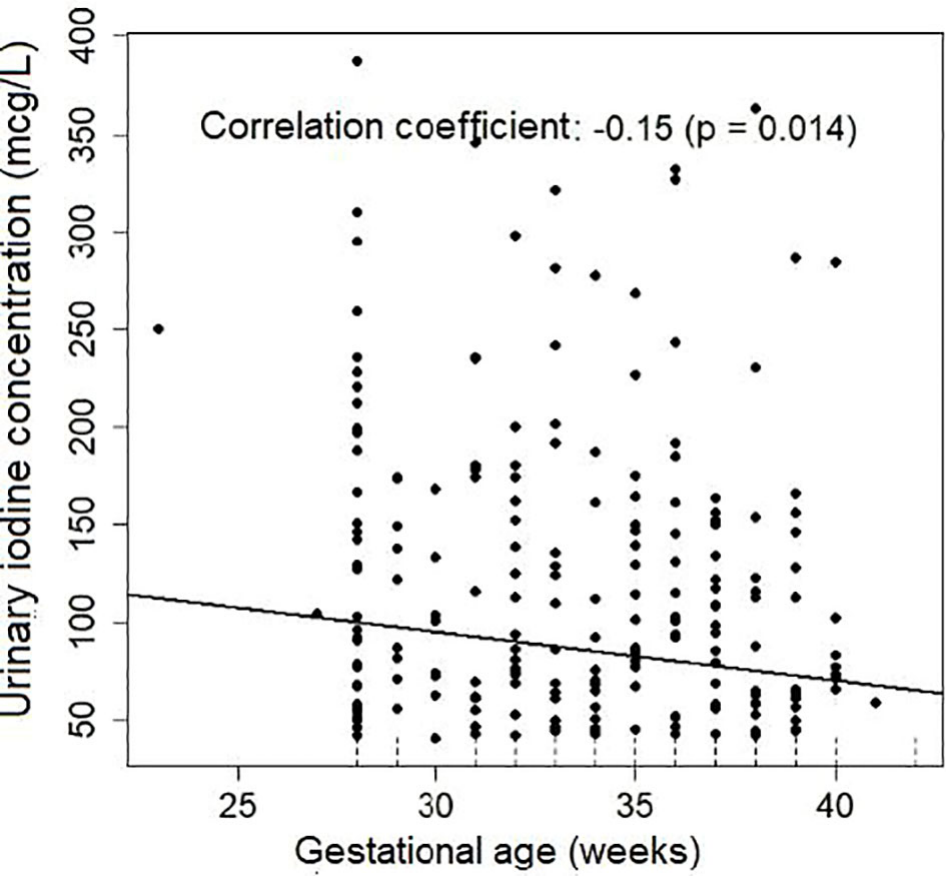
UIC correlation with gestational age.

**Figure 4:**
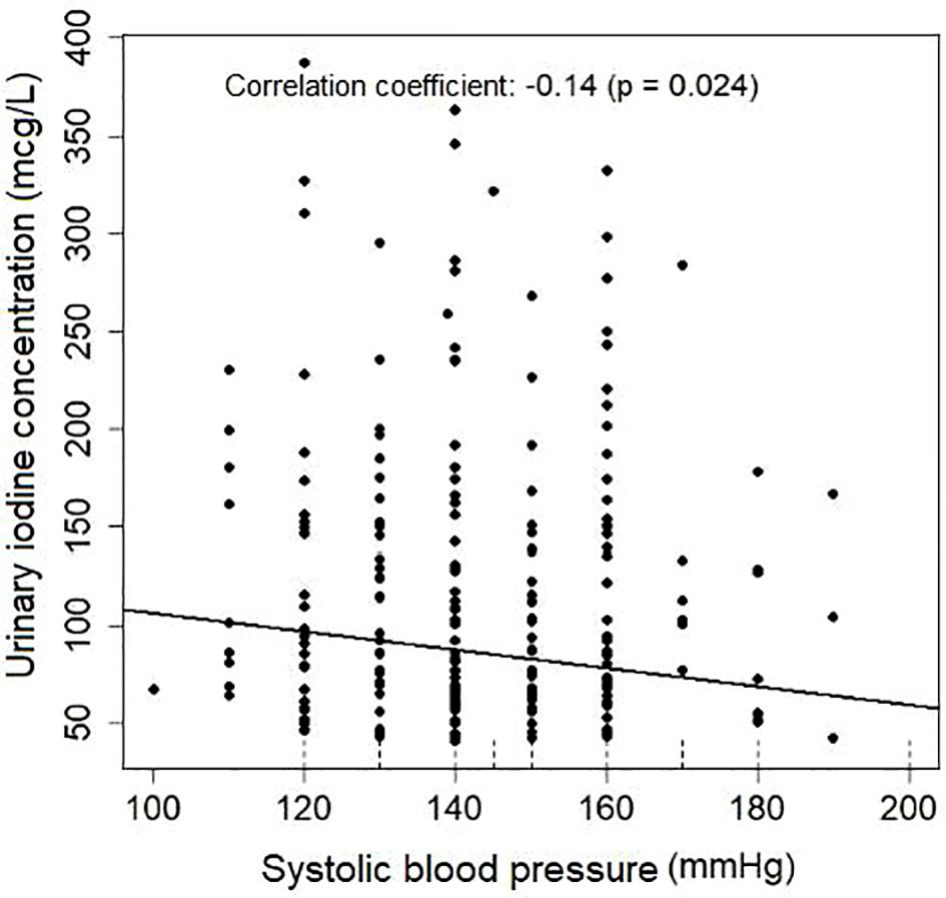
UIC correlation with systolic blood pressure.

A significant correlation was found between UIC and gestational age, BMI, and systolic blood pressure (SBP) in the initial model, without taking the type of hypertensive disorder into account. Following multivariate analysis, only gestational age (p=0.042) and SBP (p=0.015) remained significant. UIC decreased as gestational age and SBP increased (**Table 4**, **Figures 3**, **4**).

**Table 4:** Univariate and multivariate analyses of the association between urinary iodine concentration (UIC) and the clinical and laboratory characteristics.

		Univariate model		Final multivariate model	
Characteristic	n	Coefficient ± Standard Error	p-value	Coefficient ± Standard Error	p-value
Age (years)	250	0.01 ± 0.006	0.053	–	–
Gestational age (weeks)	250	-0.03 ± 0.01	0.014	-0.03 ± 0.01	0.042
Number of pregnancies	250	0.05 ± 0.03	0.096	–	–
Body mass index	250	0.02 ± 0.007	0.036	–	–
Systolic blood pressure	250	-0.006 ± 0.002	0.024	-0.006 ± 0.002	0.015
Diastolic blood pressure	250	-0.007 ± 0.004	0.060	–	–
Thyroid-stimulating hormone (TSH)	238	0.009 ± 0.041	0.827	–	–
Anti-thyroid peroxidase (anti-TPO) antibody	238	0.001 ± 0.002	0.626	–	–

Models adjusted using the cenreg function of the NADA (Nondetects and Data Analysis for Environmental Data) package of the R software, version 4.4.1.

When types of hypertensive disorders were included in the correlation analysis, the initial model showed a statistically significant correlation with gestational age, preeclampsia and superimposed preeclampsia. Following the multivariate analysis, gestational age (p=0.002), preeclampsia (p<0.001), superimposed preeclampsia (p=0.001) and eclampsia (p=0.038) were statistically significant (**Table 5**).

**Table 5:** Univariate and multivariate analyses of the association between urinary iodine concentration (UIC) and clinical and laboratory characteristics, controlling for the possible effect of hypertensive disorders of pregnancy.

		Initial multivariate model		Final multivariate model	
Characteristic	n	Coefficient ± standard error	p-value	Coefficient ± standard error	p-value
Age (years)	250	0.005 ± 0.007	0.464		
Gestational age (weeks)	250	-0.038 ± 0.012	0.001	-0.035 ± 0.011	0.002
Number of pregnancies	250	0.012 ± 0.032	0.722		
Body mass index (kg/m^2^)	250	0.013 ± 0.008	0.093		
Systolic blood pressure	250	-0.004 ± 0.004	0.260		
Diastolic blood pressure	250	0.004 ± 0.005	0.470		
Thyroid-stimulating hormone (TSH)	238	0.034 ± 0.039	0.383		
Anti-thyroid peroxidase antibody (anti-TPO)	238	0.0002 ± 0.002	0.905		
Gestational hypertension (reference)	78	–	–		
Preeclampsia	80	-0.418 ± 0.116	< 0.001	-0.486 ± 0.107	< 0.001
Superimposed preeclampsia	40	-0.444 ± 0.142	0.002	-0.429 ± 0.131	0.001
Chronic hypertension	29	-0.181 ± 0.151	0.231	-0.118 ± 0.144	0.411
Eclampsia	15	-0.339 ± 0.195	0.083	-0.392 ± 0.189	0.038
HELLP syndrome	8	-0.249 ± 0.254	0.326	-0.283 ± 0.247	0.252

HELLP syndrome: Syndrome of hemolysis, elevated liver enzymes, and low platelet count.

Models adjusted using the cenreg function of the NADA (Nondetects and Data Analysis for Environmental Data) package of the R software, version 4.4.1.

## 4 Discussion

In this study, the median UIC was 82.5 μg/L, with 76.4% of participants showing iodine deficiency. The lowest median UIC was found in the women with preeclampsia (61.3 μg/L) and superimposed preeclampsia (70.6 μg/L). No correlation was found between UIC and TSH or anti-TPO antibodies. UIC decreased as gestational age and SBP increased.

Iodine has antioxidant properties, and pregnant women with hypertensive syndromes and iodine deficiency have higher markers of oxidative stress and low antioxidant status. Iodine deficiency is believed to predispose women to preeclampsia by reducing the antioxidant capacity of the placenta, leading to increased production of free radicals and endothelial dysfunction (21).

A systematic review of studies across Africa, Europe and South America determined the iodine status in pregnant women and the risk of preeclampsia due to iodine deficiency (9). A Norwegian study involving 2,795 pregnant women reported a median UIC of 121 μg/L and an increased risk of preeclampsia in iodine-deficient women, while iodine supplementation lowered the incidence of preeclampsia (22). Nevertheless, other studies failed to confirm this association (3, 23, 24).

The highest frequency of iodine deficiency was found in the 25–34 age group (44%; n=51), however, the lowest median UIC was detected in pregnant women under the age of 25 (68.8 μg/L; 95%CI 55.7-79.6 μg/L; p=0.051). Pregnant women under the age of 25 are at greater risk of eclampsia and severe preeclampsia in late pregnancy (17), and this finding could be related to this association (21, 22). Although the age group over 35 years is also considered a risk factor for the development of preeclampsia (18, 25, 26), these pregnant women had the highest median UIC in the sample (90.1 μg/L; 95% CI 73.8-115.5 μg/L). We believe that the low number of participants in this group (27.2%; n=68) may have influenced this analysis.

The lower UIC observed in this study correlated with higher gestational age, possibly due to increased renal clearance during pregnancy, particularly from the second trimester onwards, leading to iodine depletion (27). Previous studies have reported similar reductions in UIC as pregnancy progresses (28–30), with varying consequences depending on the trimester of pregnancy (31). A study in Thailand found lower UIC levels in the second trimester, which increased the risk of preeclampsia, fetal growth restriction, low birthweight and prematurity (32). However, a UK study that analyzed the impact of iodine deficiency in all three trimesters of pregnancy found no difference in the incidence of preeclampsia, hypertension, gestational diabetes, prematurity, small-for-gestational-age fetuses or postpartum hemorrhage (3).

In this study, SBP was associated with lower UIC levels, possibly due to reduced sodium intake. A study with 241 pregnant women who followed a low-sodium diet found a 112% increased risk of iodine deficiency, suggesting that pregnant women on such a diet may require iodine supplementation (14). New studies would be necessary to specifically analyze this hypothesis.

Although we did not have a control group and considering that the northeastern region of Brazil is not iodine deficient, iodine deficiency was found in 76.4% of pregnant women with hypertensive disorders. Furthermore, the correlation between CUI, SBP and the severity of hypertensive disorders suggests a possible association between iodine deficiency and hypertensive disorders of pregnancy.

Recent studies on the association between iodine status during pregnancy and maternal and neonatal thyroid function have yielded conflicting results, with most of these studies having excluded women with hypertensive disorders (11, 27, 33–40). While severe iodine deficiency and excess iodine intake are linked to thyroid dysfunction, mild to moderate deficiency generally does not affect thyroid functions (27, 34).

This study found no correlation between UIC and TSH values. Spina et al. (11) evaluated 123 pregnant women with a median UIC of 108 μg/L and found no significant TSH abnormalities during the different trimesters of pregnancy. Similarly, a Chinese study (41) of 450 pregnant women (150 in each trimester) found no correlation between UIC and TSH or free triiodothyronine (T3) levels. However, other studies have reported a correlation between UIC and TSH (35–38, 41). A prospective study monitoring 212 women from prenatal visits until postpartum found a positive correlation between UIC and TSH levels and an association with low birthweight (42).

Physiologically, the thyroid gland can adapt to functional abnormalities by regulating the hypothalamic-pituitary-adrenal axis and increasing iodine absorption, often leading to enlarged glands (up to 10% larger than the original size) (43, 44). However, in iodine-deficient regions, such adaptative mechanisms can fail to maintain adequate reserves of intrathyroidal iodine, resulting in its dysfunction (4, 43–45). In iodine sufficient regions such as Brazil (46), the thyroid gland may adapt easily to the increased iodine demand during pregnancy, increasing iodine uptake, reducing UIC, and maintaining physiological hormone levels. Both this study and other previous studies (11, 27, 33–35) suggest that mild to moderate iodine deficiency in pregnancy does not appear to alter maternal thyroid function.

Among the participants, only 4.6% (n=11) tested positive for anti-TPO antibodies, with a median UIC of 150.2 μg/L. No association was found between autoimmunity and UIC. However, the small number of pregnant women with positive anti-TPO in the sample limited the analysis of this variable in the study. Businge et al. (47) compared normotensive pregnant women with others with preeclampsia in the third trimester and found that the women with preeclampsia had lower UIC and higher levels of TSH and anti-TPO antibodies; however, the differences were not statistically significant. Other studies have reported a correlation between iodine levels and autoimmunity (28, 35, 48). Two studies found an association represented by a U-shaped curve between UIC and abnormal thyroid hormone levels and autoimmunity (35, 49). In China, one cross-sectional study (n=7,073 pregnant women) and one prospective study (n=4,635 pregnant women) reported that iodine deficiency was associated with higher risks of positivity for anti-TPO antibodies (28, 48). Although the role of iodine in autoimmune thyroid disease has yet to be fully clarified, the intake of high doses of iodine in iodine-deficient regions could precipitate autoimmunity by inducing inflammatory responses secondary to oxidative stress (50, 51).

### 4.1 Limitations

UIC is the most common marker for evaluating iodine status (48, 51); however, it is subject to significant intra-individual variation including urine dilution and recent dietary intake (49, 52). This study did not record data on dietary intake and, and the participants were hospitalized, which accounted for a change in their normal dietary pattern. Currently, there is an ongoing debate on the ideal index for evaluating individual iodine intake in pregnant women (49).

Although 24-hour urine collection is the gold standard for measuring UIC, it is time-consuming and prone to incomplete sampling (49, 53, 54). Single-sample urine collection, as used in this study, correlates well with 24-hour collection (53). However, urinary iodine excretion may vary throughout the day and the use of a single sample may have overestimated iodine deficiency. Therefore, to control for possible interferences in UIC measurement, other markers such as serum iodine and thyroglobulin (24, 47, 55) should be evaluated. Serum iodine measurements include both free iodine and iodine incorporated into thyroid hormones, while UIC reflects only iodine that was recently ingested and excreted through the kidneys. Therefore, theoretically, serum iodine could be advantageous in relation to urinary iodine because it would reflect the amount of iodine available for the body over a more prolonged period (56); however, further population-based studies need to be conducted to validate this parameter.

The measurement of a single urine sample for UIC is, in fact, an important methodological limitation. This approach may affect the reliability of findings, especially due to intraindividual variability (e.g., through recent intake of iodine-rich foods or hydration status) in urinary iodine excretion. This variability can cause a single sample to not accurately reflect habitual iodine intake, leading to incorrect classifications of individual or population nutritional status. To circumvent this, ideally multiple samples per individual or adjustment methods such as correction for urinary creatinine or 24-hour urine collection would be used, when feasible. However, due to budgetary constraints, the study was limited to a single measurement of this marker.

Additionally, UIC was measured only in the third trimester. While iodine levels can oscillate during pregnancy, preeclampsia and gestational hypertension tend to develop around the second trimester. Therefore, measuring UIC at multiple time points, preferably in each trimester, would provide more accurate data. Finally, there was no control group in this study, with UIC being compared between the different types of hypertensive disorder, which could have resulted in an overestimation of the effect of iodine deficiency.

Although the benefits of iodine supplementation remain uncertain in cases of mild to moderate iodine deficiency, the American Thyroid Association (ATA) guidelines recommend iodine supplementation for pregnant and lactating women, as supplementation remains important due to its known role in fetal development (25).

### 4.2 Strengths

Although this was a hospital-based study, the institute is considered a reference in the region, representative of a large portion of the state population. There is a scarcity of studies evaluating iodine status in a specific population of pregnant women with hypertensive disorders, as most of the previous studies assessed non-hypertensive pregnant women. Another strongpoint refers to the evaluation of high-risk pregnant women. This study adds to a growing data set suggesting that mild to moderate iodine deficiency does not negatively affect maternal thyroid function or autoimmunity.

### 4.3 Conclusion

Among women in the third trimester of pregnancy with hypertensive disorders, the lowest UIC was found in women with preeclampsia and superimposed preeclampsia; however, the available data are insufficient to confirm the association between iodine deficiency and the risk of preeclampsia. Despite the high prevalence of iodine deficiency, no correlation was found between UIC and thyroid function or autoimmunity. These findings indicate a need to monitor iodine status during pregnancy. Studies are required to further investigate this specific profile of pregnant women.

## Data Availability

The raw data supporting the conclusions of this article will be made available by the authors, without undue reservation.
